# Differential diagnosis of eczema and psoriasis using routine clinical data and machine learning: development of a web-based tool in a multicenter outpatient cohort

**DOI:** 10.3389/fmed.2025.1667794

**Published:** 2025-10-17

**Authors:** Ning Ding, Yinhao Li, Zheng Zhao, Xiangfu Meng, Mingqiang Sun, Xueqing Ren, Ying Wang

**Affiliations:** ^1^Department of Dermatology, Shengjing Hospital, China Medical University, Shenyang, China; ^2^College of Electronics and Information Engineering, Liaoning Technical University, Huludao, China; ^3^Department of Dermatology, Dermatology Hospital, Shenyang, China; ^4^Department of Dermatology, The First Affiliated Hospital, Dalian Medical University, Dalian, China

**Keywords:** eczema, psoriasis, machine learning, clinical decision support, web-based tool

## Abstract

**Background:**

Eczema and psoriasis are common chronic dermatoses with overlapping features, making early differential diagnosis difficult. While biopsy is the gold standard, its invasiveness and dependence on clinician expertise restrict routine application, especially in primary care. To overcome these limitations, we developed a machine learning-based diagnostic tool using routine laboratory data, enabling non-invasive, accurate, and practical differentiation between eczema and psoriasis in outpatient settings.

**Methods:**

We retrospectively analyzed clinical and routine laboratory data from 57,518 patients with eczema and psoriasis across three medical centers. Patients with confirmed diagnoses and complete laboratory records were included, while those with missing key data were excluded. Eight machine learning models were trained using data from Shengjing Hospital. Model performance was evaluated using accuracy, AUC, sensitivity, specificity, PPV, NPV, F1 score, and confusion matrix. The best-performing model, XGBoost, was externally validated on independent cohorts from two other hospitals. SHapley Additive exPlanation (SHAP) were applied to assess feature importance. Finally, a web-based tool was developed integrating the optimal model with optical character recognition (OCR) for automatic data input.

**Results:**

XGBoost demonstrated the best performance, with AUCs of 0.891, 0.830, and 0.812 for the training, internal test, and external test sets, respectively. Key predictive features included dNLR, neutrophil count, SIRI, RDW, and eosinophil count, which were consistent with known clinical patterns. The final model was deployed as an interactive web tool, allowing manual or OCR-based data input to provide real-time prediction probabilities.

**Conclusion:**

This machine learning-based diagnostic tool showed strong performance and interpretability in differentiating eczema from psoriasis using routine laboratory data. The user-friendly web interface enables rapid, non-invasive decision support in outpatient clinical settings.

## Introduction

Eczema and psoriasis are two of the most common chronic inflammatory skin diseases worldwide, affecting millions of individuals and imposing a significant burden on patients’ quality of life and healthcare systems (1, 2). Despite distinct underlying pathophysiological mechanisms, eczema and psoriasis can present with overlapping clinical features such as erythema, scaling, and pruritus, which poses challenges for accurate differential diagnosis (3, 4). Eczema is subdivided into atopic and non-atopic types. The atopic variant, which primarily affects children, involves skin inflammation mediated by T cells and Th2-type cytokines in its early stages. This type is commonly linked to IgE-mediated hypersensitivity to environmental allergens, often presenting with increased total IgE and specific IgE levels. The non-atopic type of eczema, which is more frequently observed in adults, is generally not associated with allergen-specific sensitization and often presents with normal total IgE levels, although elevated IgE can still be found in a subset of patients (5). However, studies have shown that elevated IgE levels can also be present in some non-atopic eczema patients, indicating that IgE levels alone may not be a definitive marker to distinguish between these two types (6). Pediatric atopic eczema typically affects characteristic sites such as the antecubital and popliteal fossae. However, adult eczema often presents with diverse and atypical lesion morphology and distribution, making clinical identification more challenging than that of the relatively well-defined pediatric form. Without timely and effective treatment, eczema can become chronic and recurrent, significantly impacting patients’quality of life and increasing healthcare costs (7). Psoriasis, classified into several subtypes, is dominated by plaque psoriasis, which accounts for approximately 80–90% of cases. Its hallmark is well-demarcated erythematous plaques with silvery scales (3). However, early-stage or mild psoriasis may present with atypical or subtle lesions such as small erythematous patches with minimal scaling, which may be confused with eczema or other dermatoses. Delayed or missed diagnosis of psoriasis not only delays appropriate treatment but also increases the risk of serious comorbidities, including psoriatic arthritis, cardiovascular disease, and psychological disorders like depression. Given the lack of a definitive cure for psoriasis, early diagnosis and timely intervention are crucial, as emphasized by international guidelines (8). Diagnosis of both psoriasis and eczema is primarily based on clinical presentation, dermoscopy and biopsy. However, each of these methods has certain limitations. Clinical diagnosis is inherently subjective and can be influenced by the individual clinician’s experience, leading to variability in diagnostic consistency. Dermoscopy can provide supplementary imaging information to aid in the differentiation between eczema and psoriasis. However, due to overlapping features and variations in presentation, its diagnostic accuracy remains limited, particularly in atypical cases. Despite being the gold standard, the invasive nature of biopsy imposes significant limitations on its widespread adoption due to patient compliance concerns. In addition, most county-level hospitals currently lack specialized dermatologists, and it is common for internal medicine physicians to assume dermatological responsibilities. Moreover, advanced diagnostic technologies are often inaccessible in primary healthcare facilities, further increasing the difficulty of differential diagnosis. This highlights the more pressing demand for dermatological services in township health centers. Therefore, developing an accurate, efficient, and easily accessible tool to distinguish between eczema and psoriasis is crucial for improving the quality of clinical decision-making, enhancing treatment outcomes, and ultimately benefiting patients.

Machine learning (ML) is a branch of artificial intelligence that allows computers to extract patterns from data and make predictions or decisions with limited human input. In recent years, with the growing demand for large-scale data analysis in medical research and clinical practice, the importance of ML has become increasingly prominent. Its powerful data processing capabilities provide valuable tools for medical diagnosis and decision support (9–12). Similarly, ML has attracted widespread attention in dermatology, especially in the field of image analysis, where significant advancements have been made (13–15). Numerous machine learning studies have enabled early differentiation and staging of cutaneous melanoma and non-melanoma skin cancers, demonstrating significant practical value in community and primary care settings (16–18). Deep learning is a subfield of machine learning. Vatsala Anand et al. employed deep learning techniques to classify images of seven distinct skin disease categories, including Melanoma, Vascular Lesions, Benign Keratosis – Lesions, Dermatofibroma, Melanocytic Nevi, Basal Cell Carcinoma and Actinic Keratoses, achieving high accuracy in their classification (19). However, in actual clinical applications, some patients have skin lesions in private lesions that are difficult to photograph, or the quality of images is affected by scratching and secondary infections. Moreover, image models typically require large amounts of data, high-performance hardware, and privacy protection issues. In contrast, basic laboratory test data, which can be easily obtained from outpatient settings, can be readily integrated into hospital systems or online auxiliary diagnostic platforms. Machine learning models incorporating serological markers and clinical features have been increasingly utilized across various medical specialties for differential diagnosis and prognostic evaluation. For instance, Sebastian Kraszewski et al. Effectively differentiated ulcerative colitis from Crohn’s disease based on laboratory markers (20), while Yolanda Sánchez-Carro et al. demonstrated that machine learning approaches could be utilized to predict depression diagnoses and their clinical subtypes based on immunometabolic indicators and lifestyle factors (21). Similarly, Alcazer et al. developed an XGBoost model utilizing ten routine laboratory parameters to classify three subtypes of acute leukemia (APL, ALL, AML), achieving AUCs of 0.97, 0.90, and 0.89, with an overall accuracy of nearly 99% (22). Chih-Min Tsai et al. applied demographic data and laboratory values extracted from electronic health records, which included complete blood counts, differential counts, urinalysis, and biochemical parameters, to distinguish Kawasaki disease from other febrile illnesses in children using an XGBoost model, thereby supporting early diagnosis and timely intervention (23). Furthermore, Anoeska Schipper et al. Developed a machine learning model for classifying appendicitis among patients presenting with acute abdominal pain in the emergency department. This model outperformed conventional scoring systems and demonstrated comparable or superior accuracy to emergency physicians, thereby enhancing rapid clinical decision-making (24). However, ML models based on hematological parameters for disease differentiation have been less frequently reported in dermatology. Eczema and psoriasis exhibit certain differences in hematological parameters, providing a rationale for further investigation. Against this background, we conducted a multicenter retrospective study to develop multiple ML models based on clinical features and hematological parameters, identify potential predictive factors, and build an online diagnostic tool that integrates both optical character recognition (OCR) technology and manual data entry. This tool is intended to provide clinicians with a practical and efficient decision support platform.

This study investigates the differential diagnosis between eczema and psoriasis, the main contributions are summarized as follows:

1. Feature selection and data preparation: Based on clinical guidelines for eczema and psoriasis, and incorporating expert opinions from dermatologists, 31 candidate features were initially selected. After rigorous screening, 14 key features were retained. A high-quality dataset was constructed from three medical centers through systematic data cleaning, classification, and selection from a large-scale hospital-based database.
2. Model development and optimization: Eight machine learning models were developed, including k-Nearest Neighbors (KNN), Decision Tree (DT), Neural Network (NNet), Random Forest (RF), Support Vector Machine (SVM), Light Gradient Boosting Machine (LightGBM), and Extreme Gradient Boosting (XGBoost). Multiple rounds of parameter tuning were conducted, and a soft-voting ensemble model (SVEM) was created by integrating the top five models. Among them, the XGBoost model exhibited the best overall performance.
3. Model interpretation: To enhance interpretability, SHapley Additive exPlanations (SHAP) was used to identify the most influential features in the XGBoost model. These features were consistent with clinical guidelines, suggesting that the model has successfully learned key knowledge required for distinguishing between eczema and psoriasis.
4. Clinical application: An online diagnostic tool was constructed based on the final model, aiming to assist clinical diagnosis in primary care institutions. The platform allows clinicians to input routine laboratory and clinical data, either manually or through OCR technology, which allows automatic extraction of text data from images of laboratory reports, thereby reducing workload and improving diagnostic efficiency.

## Materials and methods

### Data source

For this retrospective cohort study, we included patients with the diagnosis of eczema or psoriasis who attended the dermatology outpatient departments of Shengjing Hospital of China Medical University, Shenyang Dermatology Hospital, and the First Affiliated Hospital of Dalian Medical University between January 10, 2019 and January 10, 2025. All three hospitals are tertiary general hospitals directly managed by the National Health Commission of China, ensuring the generalizability and reliability of the data. This study was approved by the Ethics Committee of Shengjing Hospital of China Medical University (approval number: 2025PS1210K). Authorized physicians accessed the outpatient electronic systems to identify all patients diagnosed with “eczema” or “psoriasis” from January 10, 2019 to January 10, 2025. Clinical and laboratory data for these patients were then extracted for analysis. This cohort was subsequently screened according to the following exclusion criteria: (1) patients with incomplete hematological parameters and basic information; (2) age <18; (3) patients with other concomitant skin diseases; (4) patients with other systemic diseases such as hypertension, diabetes, or coronary heart disease; (5) non-first-time visitors; (6) patients who used medications on their own before the visit.

### Feature selection

A total of 31 candidate variables, comprising demographic characteristics, standard hematological characteristics obtained from complete blood count (CBC), and derived inflammatory markers were initially collected. Hematological characteristics included white blood cell count (WBC); percentages and absolute counts of neutrophils, lymphocytes, monocytes, eosinophils, and basophils; red blood cell count (RBC); hemoglobin (HGB); hematocrit (HCT); mean corpuscular volume (MCV); mean corpuscular hemoglobin (MCH); mean corpuscular hemoglobin concentration (MCHC); red cell distribution width (RDW); platelet count (PLT); plateletcrit (PCT); mean platelet volume (MPV); platelet distribution width (PDW); and total IgE levels. Derived inflammatory indices included the neutrophil-to-lymphocyte ratio (NLR), derived NLR (dNLR), monocyte-to-lymphocyte ratio (MLR), neutrophil-plus-monocyte-to-lymphocyte ratio (NMLR), systemic nflammation response index (SIRI), systemic immune-inflammation index (SII), and hemoglobin-to-red blood cell ratio (HRR) (25–27). Prior to analysis, all variables underwent integrity and consistency checks. Records containing any missing values were excluded. Categorical variables were factorized and encoded as dummy variables. Specifically, gender was encoded as 0 for female and 1 for male, while disease type was encoded as 0 for eczema and 1 for psoriasis. To reduce the impact of extreme values on model performance, outliers exceeding three standard deviations from the mean were removed. All 31 variables were subjected to feature selection using the Boruta algorithm, a robust and widely used wrapper method based on random forest classification. Boruta assesses the importance of each variable by creating “shadow features,” which are randomized copies of the original variables, and then comparing the Z-scores of the actual variables with those of the shadow features. If a variable consistently exhibits a significantly higher Z-score than the maximum among its shadow features across multiple iterations, it is deemed “important” and retained for model construction. Otherwise, it is labeled “unimportant” and excluded (28). This process allows the algorithm to identify features that meaningfully contribute to model performance, even in the presence of complex and nonlinear relationships. Notably, Boruta focuses on the overall relevance of each variable within the model context, meaning that variables showing significance in univariate analysis may still be excluded if their predictive contribution is limited (29). After feature selection, Spearman correlation analysis was performed to assess multicollinearity among the selected variables. While most machine learning algorithms are relatively robust to multicollinearity, it can still affect the interpretation of feature importance. When two variables were highly correlated (defined as a Spearman correlation coefficient >0.7), one was excluded based on clinical relevance or statistical contribution (30). The final set of independent variables was determined in conjunction with expert advice from dermatologists.

### Model construction and evaluation

After applying the inclusion and exclusion criteria, eligible patient data from Shengjing Hospital were randomly divided into a training set and an internal test set at a 6:4 ratio. To assess model generalizability, an external test set was constructed using data from 916 patients collected at Shenyang Dermatology Hospital and the First Affiliated Hospital of Dalian Medical University. The 14 predictive variables selected in the previous step were used as input features. Seven machine learning models were applied, including KNN, DT, NNet, RF, SVM, LightGBM, and XGBoost (31–33). In addition, SVEM was developed as the eighth model by combining the probabilistic outputs of the five best-performing classifiers using weighted averaging (34, 35). This ensemble approach aimed to leverage the complementary strengths of different algorithms to enhance robustness and reduce overfitting. All models were trained using 10-fold cross-validation on the training set. To achieve optimal model performance, hyperparameters were tuned with the aim of maximizing the area under the receiver operating characteristic curve (AUC). In addition to AUC, model performance was evaluated on both internal and external test sets using multiple metrics, including confusion matrix, accuracy, sensitivity (recall), specificity, positive predictive value (PPV), negative predictive value (NPV), and F1-score. To improve the interpretability of the model, we applied SHAP to produce dependence plots that visualize the individual contribution and influence of each feature on the prediction outcomes (36). All analyses were conducted using R software (version 4.4. 1).

### Machine learning model

This study employed eight representative machine learning algorithms for model development, including KNN, DT, NNet, RF, SVM, LightGBM, XGBoost and SVEM. Brief introductions to each classifier are as follows.

1. KNN: K-Nearest Neighbors is a non-parametric, instance-based supervised learning algorithm. It classifies data points by calculating distances and selecting the majority class among the k-nearest neighbors in the feature space. Its simplicity and interpretability make it suitable for small datasets with low dimensionality and well-separated classes (37).
2. DT: Decision Tree is a supervised learning algorithm that recursively splits data based on feature values, forming a tree-like structure for classification or regression tasks. Each internal node represents a decision based on a feature, and the leaves correspond to class labels. It is highly interpretable and effective for capturing non-linear relationships (38).
3. NNet: Neural Networks are computational models inspired by biological neural systems, composed of layers of interconnected nodes (neurons). They are capable of learning complex patterns and are highly adaptable to various types of data, forming the foundational architecture for many deep learning methods (39).
4. RF: Random Forest is an ensemble learning method that constructs multiple decision trees and merges their results to improve accuracy and control overfitting. It handles large datasets with higher dimensionality and provides estimates of feature importance (40).
5. SVM: Support Vector Machine constructs optimal separating hyperplanes in high-dimensional spaces to distinguish between classes with maximum margin. It is highly effective for small sample sizes and high-dimensional data, and can handle non-linear problems through the use of kernel functions (41).
6. LightGBM: Light Gradient Boosting Machine is a gradient boosting framework that uses tree-based learning algorithms, designed for speed and efficiency. It offers faster training speed and lower memory usage. It employs a histogram-based decision tree algorithm and a leaf-wise growth strategy to enhance computational efficiency. The model supports native handling of categorical features and enables efficient multi-threaded training. LightGBM is particularly suitable for large-scale, high-dimensional datasets requiring fast and accurate learning (42).
7. XGBoost: Extreme Gradient Boosting is an advanced and efficient implementation of the gradient boosting framework, specifically optimized for computational speed and model performance. It incorporates regularization techniques to reduce overfitting, supports parallel processing to accelerate training, and is capable of handling missing values natively. Due to its high predictive accuracy and robustness, XGBoost has been widely adopted in both academic research and practical machine learning applications (43).
8. SVEM: The Soft Voting Ensemble Model combines the predicted probabilities from multiple base classifiers and performs weighted averaging to determine the final class. By leveraging the complementary strengths of different models, it enhances predictive performance, improves generalizability, and reduces the risk of overfitting compared to individual classifiers (34, 35).

### Model evaluation indices

To comprehensively evaluate the model’s ability to discriminate between patients with eczema (defined as the negative class) and those with psoriasis (defined as the positive class), we applied several evaluation metrics including the confusion matrix, AUC, accuracy, sensitivity, specificity, PPV, NPV, and F1 score (44, 45). The definitions and corresponding formulas are provided below.

#### Confusion matrix

The confusion matrix is a 2 × 2 table that compares predicted and actual class labels. It includes:

- TP (True positive): Psoriasis cases correctly identified.
- FP (False positive): Eczema cases incorrectly predicted as psoriasis.
- TN (True negative): Eczema cases correctly identified.
- FN (False negative): Psoriasis cases incorrectly predicted as eczema.

**Table:** 

	Actual positive (psoriasis)	Actual negative (eczema)
Predicted positive	TP	FP
Predicted negative	FN	TN

#### Accuracy

$$\;\text{Accuracy}=\frac{\mathrm{TP}+\mathrm{TN}}{\mathrm{TP}+\mathrm{TN}+\mathrm{FP}+\mathrm{FN}}$$

Reflects the overall proportion of correct predictions.

#### Sensitivity/recall

$$\text{Sensitivity}=\frac{\mathrm{TP}}{\mathrm{TP}+\mathrm{FN}}$$

Measures the model’s ability to correctly identify psoriasis cases, reflecting its capability to minimize false negatives.

#### Specificity

$$\text{Specificity}=\frac{\mathrm{TN}}{\mathrm{TN}+\mathrm{FP}}$$

Indicates the model’s ability to correctly identify eczema cases, reflecting its capability to minimize false positives.

#### Positive predictive value (PPV)/precision

$$\mathrm{PPV}=\frac{\mathrm{TP}}{\mathrm{TP}+\mathrm{FP}}$$

Represents the proportion of true psoriasis cases among all predicted positive cases, reflecting the accuracy of positive predictions.

#### Negative predictive value (NPV)

$$\mathrm{NPV}=\frac{\mathrm{TN}}{\mathrm{TN}+\mathrm{FN}}$$

Represents the proportion of true eczema cases among all predicted negative cases, reflecting the accuracy of negative predictions.

#### F1 score

$$F1=2\times \frac{\text{Precision}\times \text{Recall}\;}{\text{Precision}+\text{Recall}}$$

The F1 score is the harmonic mean of precision (positive predictive value) and recall (sensitivity). It is particularly useful in evaluating the model’s ability to diagnose psoriasis when both false positives and false negatives need to be minimized. The F1 score provides a balanced measure that is especially valuable in cases of class imbalance.

#### Area under the curve (AUC)

AUC represents the area under the receiver operating characteristic curve and reflects the model’s overall ability to discriminate between eczema and psoriasis. A higher AUC indicates better classification performance and is robust to class imbalance, making it one of the most important metrics for differential diagnosis in this study.

### Web deployment of the model

The final prediction model is intended to be implemented as an online web application. When users input the relevant features, the system will generate a prediction indicating whether the patient is more likely to have psoriasis or eczema, along with the corresponding probability score. To enhance usability and reduce the time burden on clinicians, an OCR function has been integrated, allowing users to either enter data manually or upload laboratory reports for automated extraction of relevant information.

## Results

### Data resource

**Figure 1:**
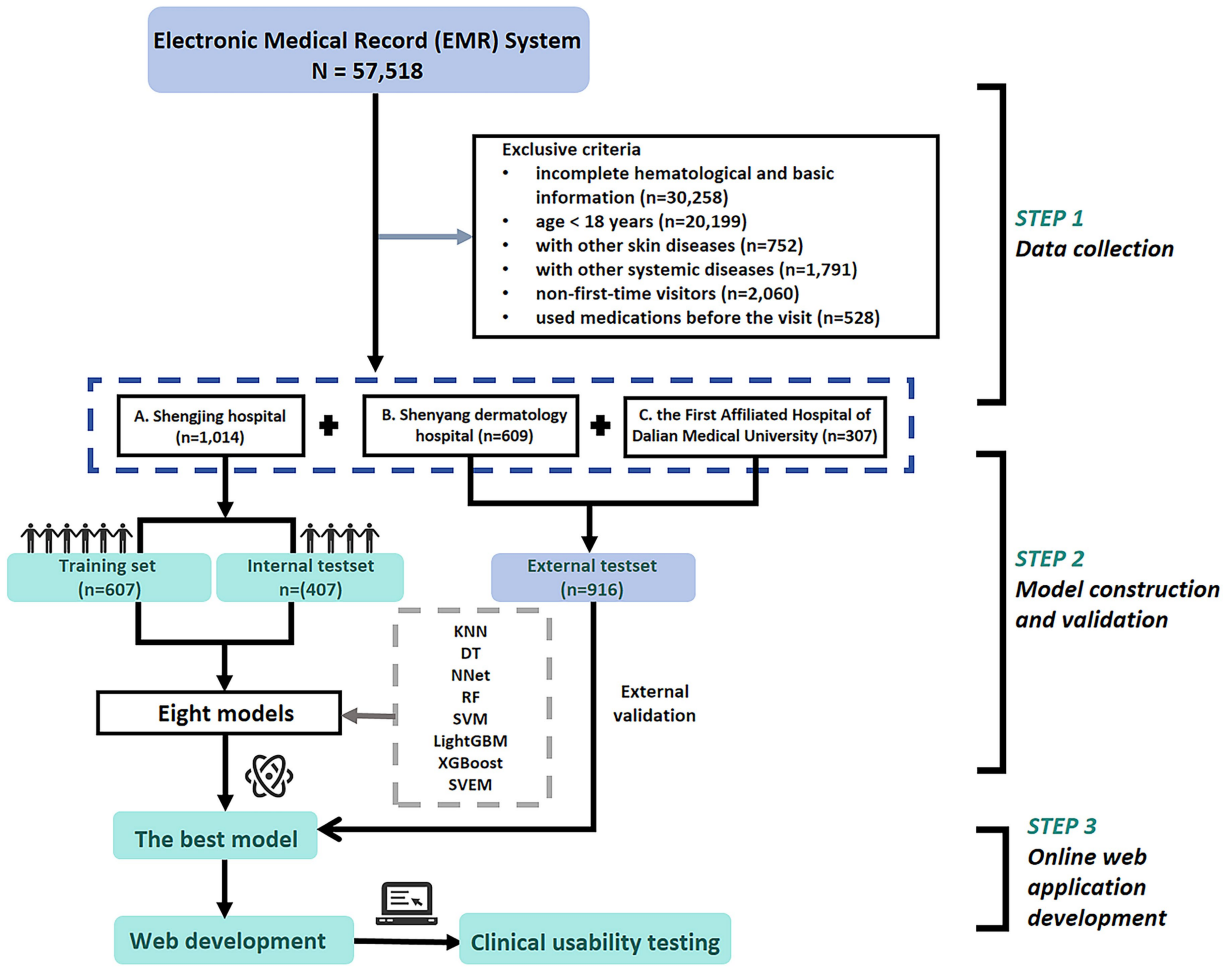
Patient flowchart and study design.

After applying inclusion and exclusion criteria, a total of 1,014 patients were selected from 29,872 cases at Shengjing Hospital, including 541 cases of eczema and 473 cases of psoriasis. Additionally, an external validation cohort consisting of 916 patients (485 eczema and 431 psoriasis) was selected from 27,646 cases at Shenyang Dermatology Hospital and the First Affiliated Hospital of Dalian Medical University. The detailed cohort selection process is illustrated in Figure 1.

### Feature selection

A total of 1,014 patients from Shengjing Hospital were included for initial analysis using the full dataset. The normality of continuous variables was evaluated with the Shapiro–Wilk test. Normally distributed variables were compared by independent samples t-tests and expressed as mean ± standard deviation (Mean ± SD). For variables not normally distributed, the Mann–Whitney U test was applied, and results were reported as median and interquartile range (Medians and IQRs). Categorical data were analyzed using the chi-square test. The baseline characteristics are shown in Table 1, indicating that 16 out of 31 variables significantly differed between patients with psoriasis and those with eczema.

**Table 1:** Baseline characteristics of the study population.

Variable category	Variables	Total (*n* = 1,014)	Psoriasis (*n* = 473)	Eczema (*n* = 541)	*p* value
Personal history	Age	38 (29, 54)	37 (28, 50)	41(30, 59)	**<0.01**
	Gender				
	Male	466 (45.96)	221 (46.72)	245 (45.29)	0.65
	Female	548 (54.04)	252 (53.28)	296 (54.71)	
Clinical blood tests	WBC, 10^9/L	6.74 (5.70, 8.15)	6.95 (5.93, 8.33)	6.60 (5.56, 7.91)	**<0.01**
	NeutPercent, %	60.48 ± 8.97	61.79 ± 8.81	59.34 ± 8.96	**<0.01**
	LymphPercent, %	29.32 ± 8.42	29.06 ± 7.88	29.56 ± 8.87	0.34
	MonoPercent, %	6.30 (5.20, 7.70)	6.40 (5.30, 7.70)	6.20 (5.20, 7.70)	0.26
	EosPercent, %	2.00 (1.10, 3.60)	1.40 (0.90, 2.40)	2.80 (1.60, 4.80)	**<0.01**
	BasoPercent, %	0.50 (0.30, 0.70)	0.50 (0.30, 0.70)	0.40 (0.30, 0.60)	**<0.01**
	NeutCount, 10^9/L	4.10 (3.30, 5.10)	4.30 (3.50, 5.40)	3.90 (3.10, 4.90)	**<0.01**
	LymphCount, 10^9/L	1.90 (1.50, 2.40)	2.00 (1.60, 2.40)	1.90 (1.50, 2.30)	0.05
	MonoCount, 10^9/L	0.40 (0.30, 0.50)	0.40 (0.40, 0.60)	0.40 (0.30, 0.50)	**<0.01**
	EosCount, 10^9/L	0.13 (0.08, 0.25)	0.10 (0.06, 0.17)	0.19 (0.10, 0.31)	**<0.01**
	RBC, 10^12/L	4.71 (4.40, 5.15)	4.78 (4.40, 5.10)	4.70 (4.40, 5.15)	0.73
	HGB, g/L	143.00 (133.00, 156.00)	143.00 (134.00, 156.00)	143.00 (133.00, 156.00)	0.35
	HCT, %	43.00 (39.80, 46.30)	43.10 (40.10, 46.20)	42.70 (39.50, 46.30)	0.24
	MCV, fL	90.00 (88.00, 93.00)	91.00 (88.00, 93.00)	90.00 (87.40, 92.40)	**<0.01**
	MCH, pg	30.30 (29.30, 31.20)	30.40 (29.40, 31.30)	30.20 (29.30, 31.20)	0.07
	MCHC, g/L	335.00 (330.00, 340.00)	335.00 (330.00, 340.00)	336.00 (330.00, 341.00)	0.47
	RDW, %	13.00 (12.60, 13.50)	13.10 (12.70, 13.60)	12.90 (12.50, 13.40)	**<0.01**
	PLT, 10^9/L	246.00 (206.00, 288.00)	244.00 (208.00, 288.00)	248.00 (205.00, 287.00)	0.50
	MPV, fL	8.80 (8.10, 9.58)	8.40 (7.80, 9.30)	9.10 (8.30, 9.70)	**<0.01**
	PCT, %	0.21 (0.18, 0.25)	0.21 (0.18, 0.24)	0.22 (0.19, 0.26)	**<0.01**
	PDW, %	16.50 (16.10, 16.90)	16.60 (16.20, 17.00)	16.30 (16.00, 16.70)	**<0.01**
	IgE	63.47 (23.70, 196.50)	49.15 (17.94, 129.00)	85.70 (31.96, 269.30)	**<0.01**
Derived inflammatory indices*	NLR	2.10 (1.58, 2.76)	2.14 (1.64, 2.84)	2.07 (1.54, 2.69)	0.10
	dNLR	0.87 (0.83, 0.89)	0.88 (0.85, 0.90)	0.86 (0.81, 0.89)	**<0.01**
	MLR	0.22 (0.17, 0.29)	0.23 (0.17, 0.29)	0.21 (0.17, 0.29)	0.36
	NMLR	2.33 (1.78, 3.00)	2.38 (1.82, 3.10)	2.29 (1.74, 2.96)	0.11
	SIRI	0.89 (0.62, 1.34)	0.95 (0.68, 1.41)	0.84 (0.57, 1.29)	**<0.01**
	SII	520.45 (367.79, 697.10)	528.75 (380.27, 748.64)	505.89 (360.21, 671.05)	0.07
	HRR	11.07 (10.22, 12.13)	10.98 (10.14, 12.10)	11.15 (10.29, 12.15)	0.31

*NLR, Neutrophil-lymphocyte ratio; dNLR, Derived neutrophil-to-lymphocyte ratio; MLR, Monocyte-to-lymphocyte ratio; NMLR, Neutrophil-to-monocyte-plus-lymphocyte ratio; SIRI, Systemic inflammation response index; SII, Systemic immune-inflammation index; HRR, Hemoglobin-to-red blood cell distribution width ratio. Bold values indicate statistical significance at *p* < 0.05.

**Figure 2:**
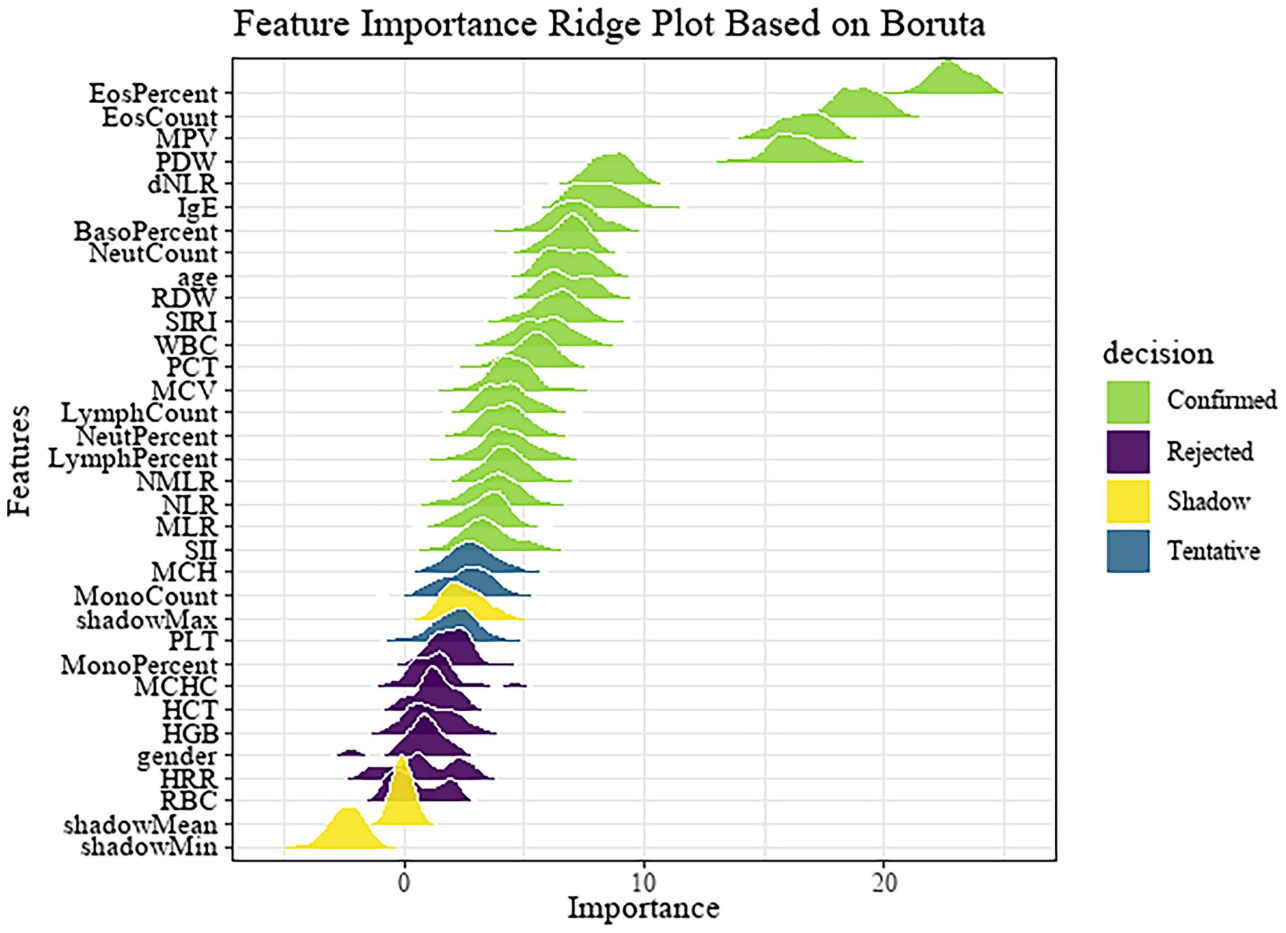
Feature importance ridge plot based on Boruta.

Subsequently, the dataset was randomly divided into a training set (*n* = 607) and a test set (*n* = 407) with a 6:4 ratio, where 60% of the data were used for model development and 40% for internal validation. Feature selection was performed exclusively on the training set to avoid data leakage. The Boruta algorithm, a wrapper method based on random forest classification, was applied to the training set to identify important features. The results are illustrated in Figure 2.

**Figure 3:**
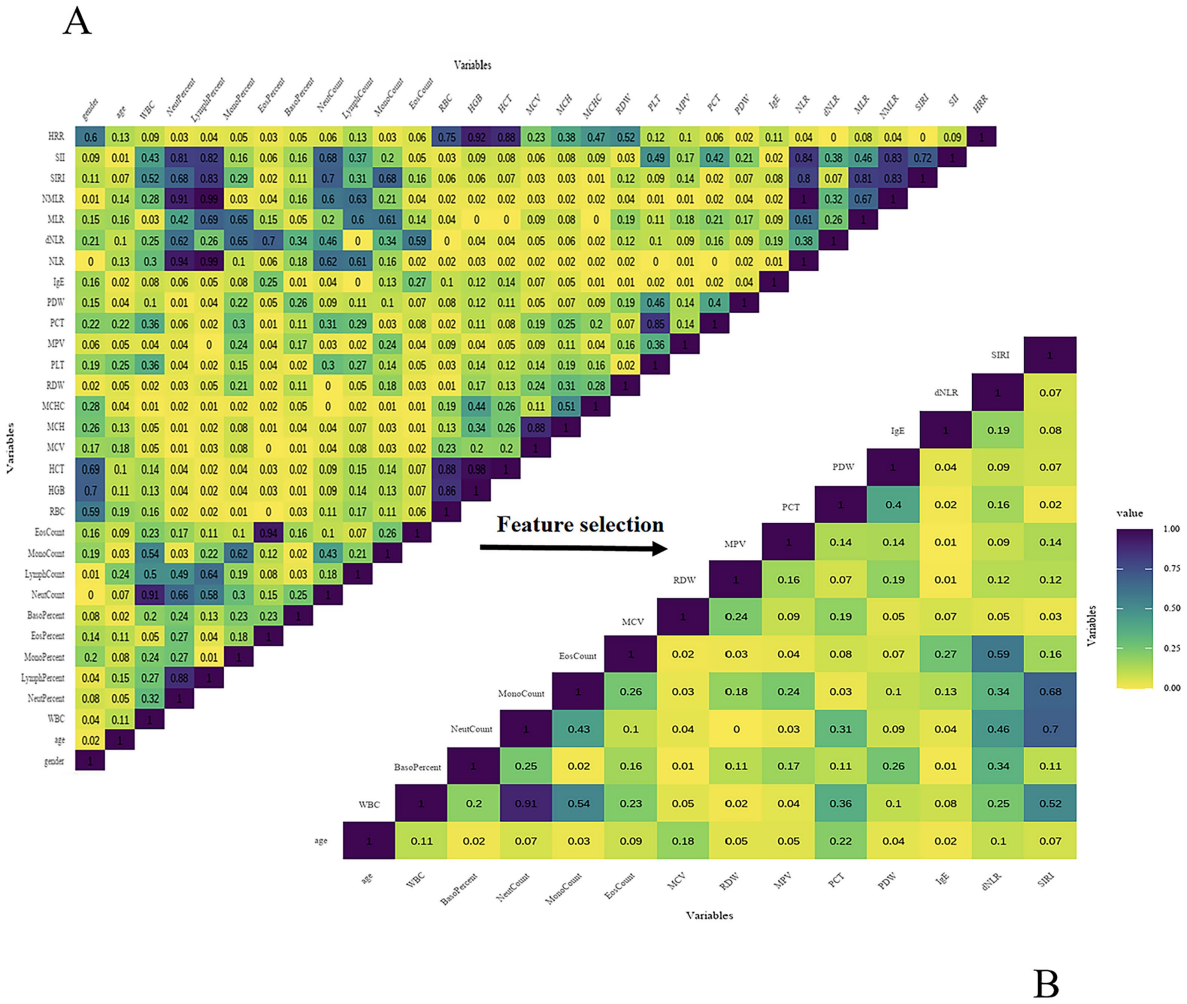
**(A)** Spearman correlation heatmap (preliminary features). **(B)** Spearman correlation heatmap (final features).

Following preliminary feature selection, Spearman correlation analysis was conducted to evaluate multicollinearity among the selected variables. The correlation heatmap is presented in Figure 3A. Based on the correlation analysis results and clinical expert opinion, the final set of selected features is shown in Figure 3B. Ultimately, 14 independent features were retained for subsequent model development, including SIRI, dNLR, IgE, PDW, PCT, MPV, RDW, MCV, EosCount, MonoCount, BasoPercent, NeutCount, WBC, age.

The baseline characteristics of the training and test sets after feature selection are shown in Supplementary Table S1, with all *p*-values greater than 0.05, indicating no statistically significant differences and confirming the adequacy of the random split.

### Model construction and validation

**Figure 4:**
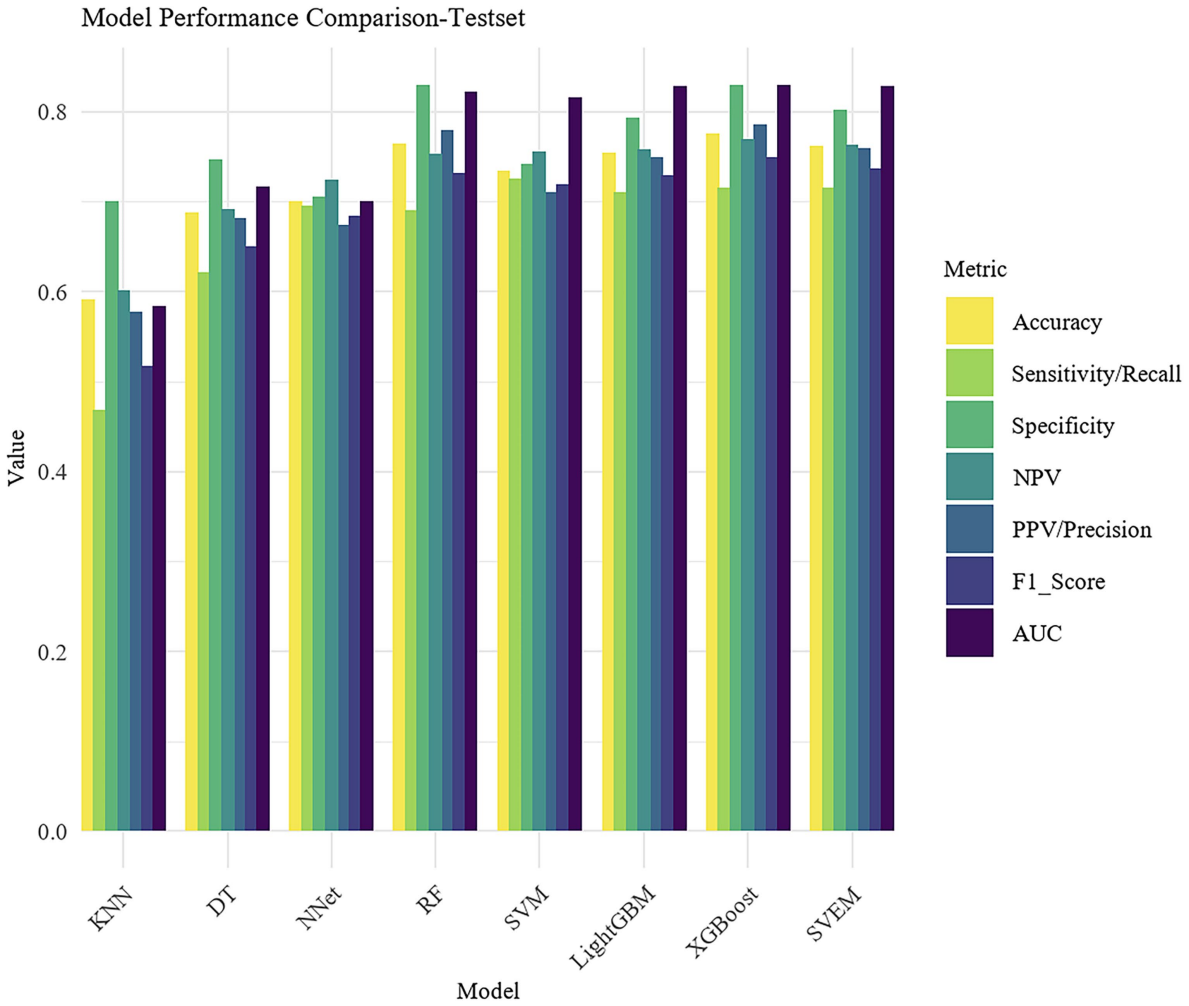
Bar plot comparison of model performance metrics.

Using 14 variables, we developed eight machine learning models, including RF, SVM, LightGBM, XGBoost, DT, KNN, NNet and SVEM. Given that psoriasis may have more severe clinical consequences and practical significance for patients, psoriasis was designated as the positive class (1) and eczema as the negative class (0) in the analysis. Table 2 presents the performance metrics, including Accuracy, Sensitivity/Recall, Specificity, PPV, NPV, F1 score, and AUC for both the training and internal test sets. To facilitate direct comparison of the models, bar plots were generated as shown in Figure 4.

**Table 2:** Performance metrics of machine learning models on the training and test sets.

Metric/Model								
Training set								
	KNN	DT	NNet	RF	SVM	LightGBM	XGBoost	SVEM
Accuracy	0.636	0.723	0.764	0.815	0.806	**0.832**	0.806	0.811
Sensitivity/Recall	0.562	0.650	0.735	0.742	**0.788**	0.781	0.728	0.749
Specificity	0.701	0.787	0.790	**0.880**	0.821	0.877	0.874	0.864
PPV/Precision	0.621	0.727	0.754	0.843	0.794	**0.847**	0.834	0.828
NPV	0.647	0.720	0.773	0.796	0.816	**0.821**	0.786	0.798
F1_Score	0.590	0.687	0.744	0.789	0.791	**0.813**	0.777	0.787
AUC	0.631	0.748	0.763	0.906	0.875	**0.904**	0.891	0.894

Testset								
	KNN	DT	NNet	RF	SVM	LightGBM	XGBoost	SVEM
Accuracy	0.592	0.688	0.700	0.764	0.735	0.754	**0.776**	0.762
Sensitivity/Recall	0.468	0.621	0.695	0.690	**0.726**	0.711	0.716	0.716
Specificity	0.701	0.747	0.705	**0.830**	0.742	0.793	**0.830**	0.802
PPV/Precision	0.578	0.682	0.674	0.780	0.711	0.750	**0.786**	0.760
NPV	0.601	0.692	0.725	0.753	0.756	0.758	**0.769**	0.763
F1_Score	0.517	0.650	0.684	0.732	0.719	0.730	**0.749**	0.737
AUC	0.584	0.717	0.700	0.822	0.816	0.828	**0.830**	0.829

Bold values indicate the best performance values for each evaluation metric.

**Figure 5:**
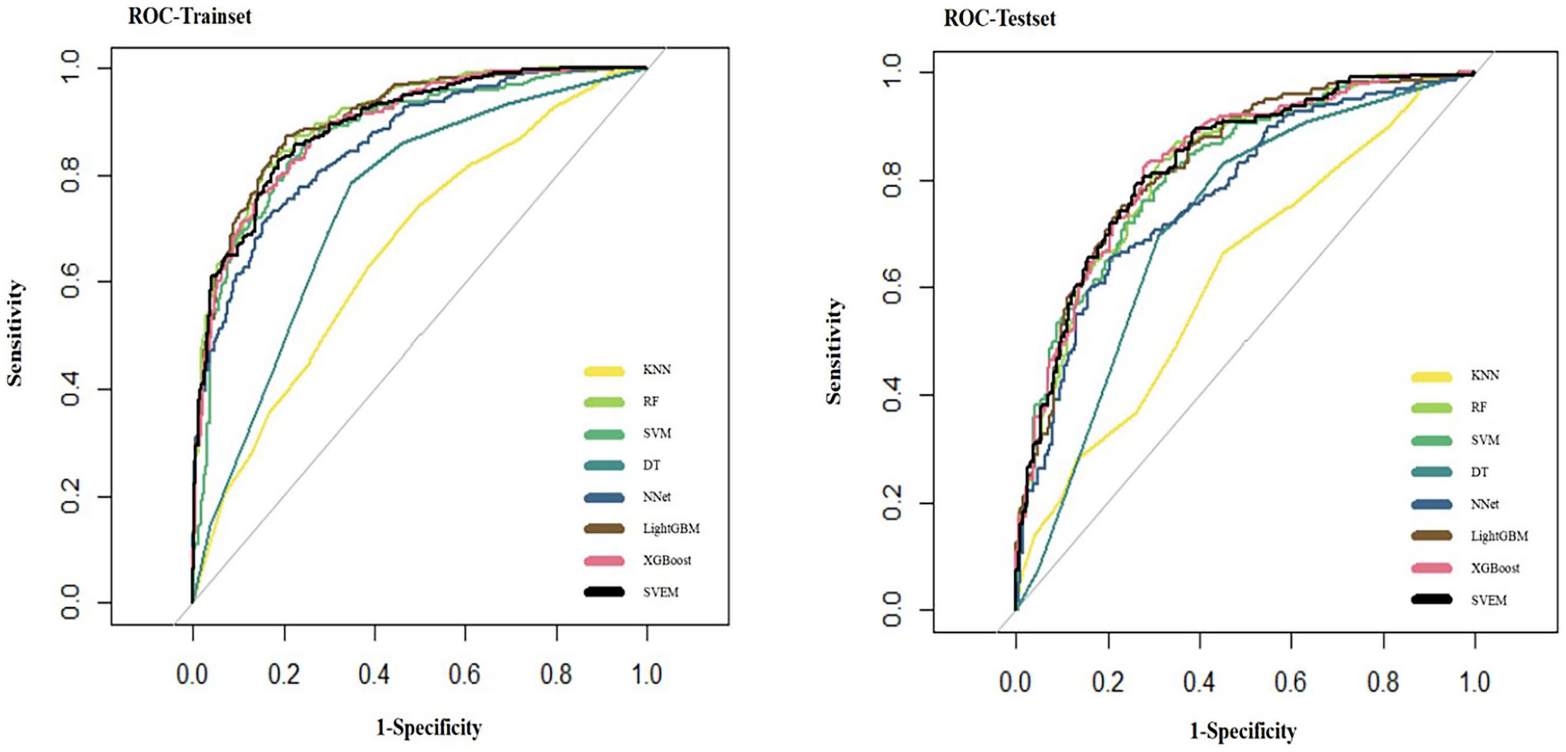
ROC curves of eight machine learning models on the internal test set.

Figure 5 presents the ROC curves for all eight ML models on the test set.

**Figure 6:**
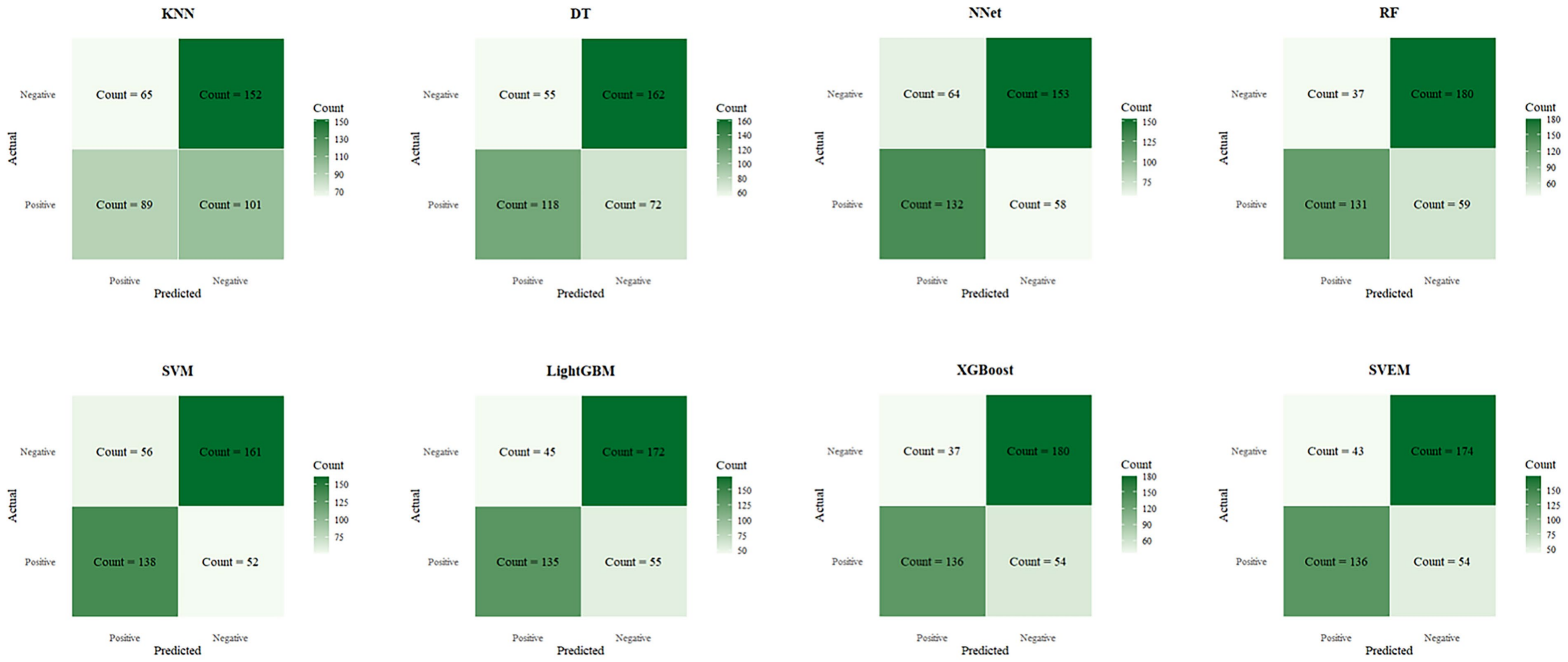
Confusion matrices of eight machine learning models on the internal test set.

Figure 6 presents the confusion matrix on the testset, which visually contrasts the performance of different models. The heatmap employs a color gradient where intensity scales with magnitude, with darker hues representing higher values.

In both the training and internal test sets, XGBoost, RF, LightGBM, SVM, and SVEM all demonstrated strong classification capabilities. The performance on the test set serves as a more reliable indicator of the model’s generalization ability, as the test data was not involved in the training process and provides a more accurate assessment of the model’s predictive power on unseen data. Therefore, the following comparisons and analyses are based on the results from the internal test set. XGBoost performed the most balanced in the internal test set, with a sensitivity of 0.716 (indicating its ability to correctly identify psoriasis patients) and a specificity of 0.830 (indicating its ability to correctly identify eczema patients). The AUC was 0.830, demonstrating the strongest overall classification ability. The AUC values for the SVEM, LightGBM, RF, and SVM were 0.829, 0.828, 0.822, and 0.816, respectively. In contrast, DT, NNet, and KNN models performed less favorably, with KNN exhibiting the lowest accuracy and AUC across all datasets. In the internal test set, KNN’s accuracy was 0.592 and AUC was 0.584, indicating its relatively weak classification capability and limited clinical application value. In summary, although different models show strengths in various metrics, XGBoost’s consistently superior performance in both the training and internal test sets makes it the optimal choice. To further assess its generalization ability, we performed external validation of XGBoost. The XGBoost model demonstrated strong performance on the external test set, with an AUC of 0.812. Together with an accuracy of 0.741, sensitivity of 0.704, specificity of 0.783, PPV of 0.742, NPV of 0.743, and an F1 score of 0.722, these results collectively confirmed the model’s robustness and generalizability in real-world clinical settings.

### Feature importance analysis

**Figure 7:**
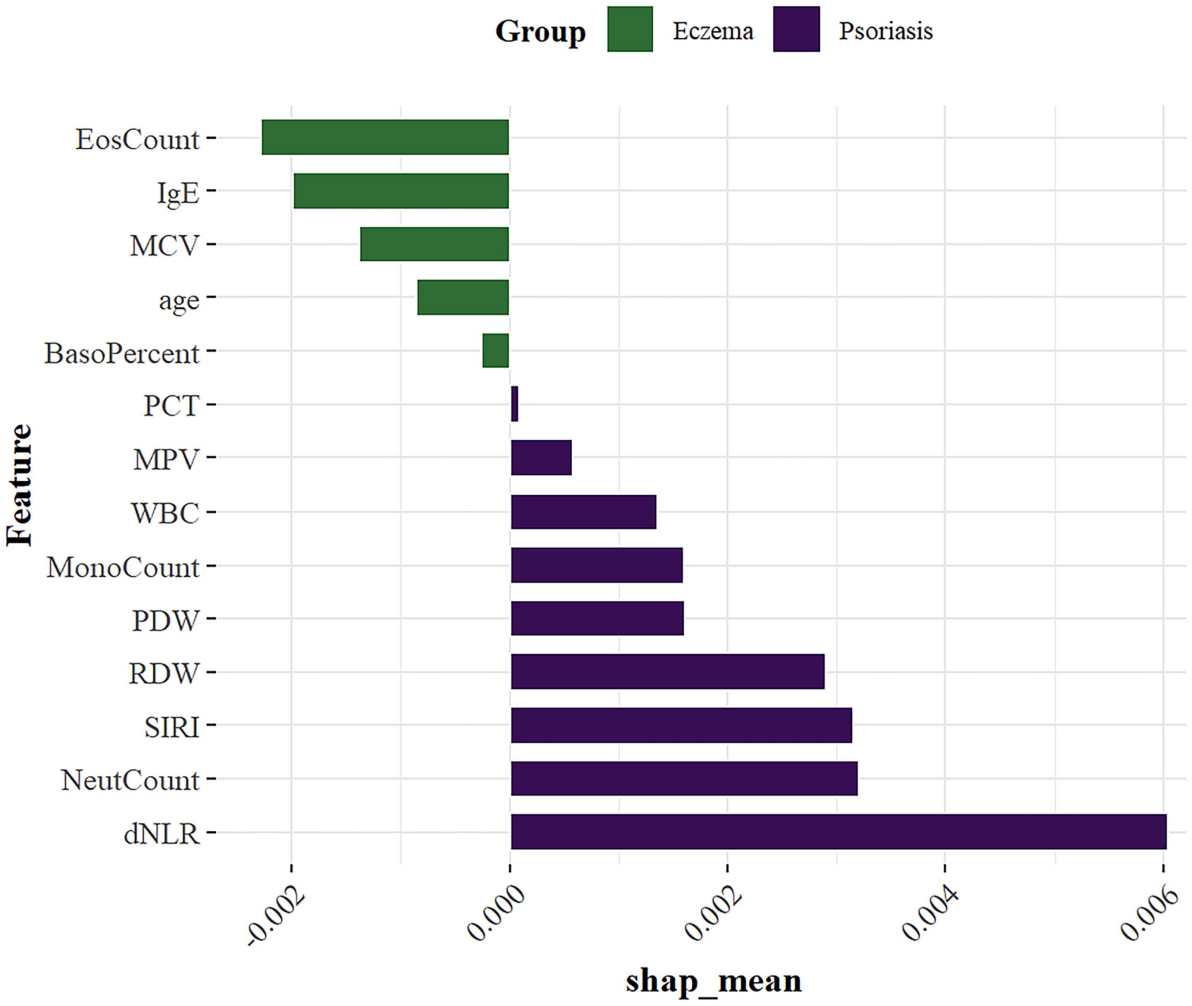
SHAP summary plot of feature importance.

According to the above results, the XGBoost model demonstrated the best performance among all candidate models, showing excellent classification ability in distinguishing between eczema and psoriasis. Although ML models are often considered ‘black boxes’ due to their lack of interpretability, this study introduced the SHAP method to perform feature importance analysis, which significantly enhanced the model’s transparency. SHAP quantifies the marginal contribution of each feature to the model’s predictions, revealing not only the overall importance through absolute SHA*p* values, but also the direction of influence (Figure 7). This helps to deepen understanding of the model’s decision-making process and expands its potential utility in clinical practice.

SHAP analysis showed that among the 14 included features, the most important variables ranked in descending order were: dNLR, NeutCount, SIRI, RDW, EosCount, IgE, PDW, MonoCount, MCV, WBC, age, MPV, BasoPercent, and PCT. These key variables help reveal potential differences between eczema and psoriasis, providing strong data support for clinical differential diagnosis.

### Web deployment of the model

**Figure 8:**
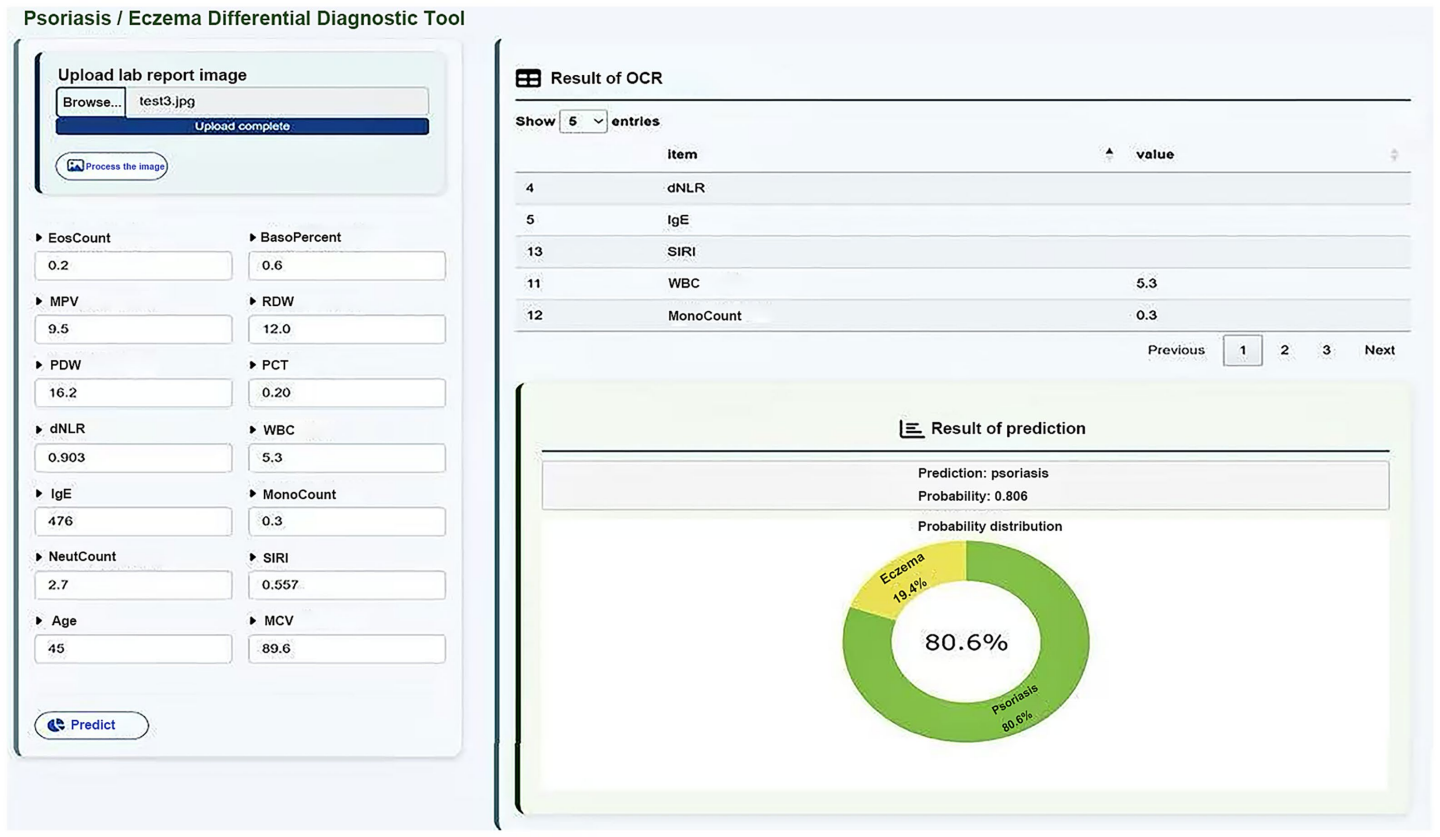
Web interface of the ML-based diagnostic system with OCR integration.

As shown in Figure 8, we developed an intelligent auxiliary diagnostic webpage that integrates machine learning with OCR technology, based on the XGBoost model. The specific operating procedure of the web-based diagnostic system is illustrated in Video 1.

The system was designed with a focus on user-friendliness and clinical applicability, supporting two modes of data entry: (1) manual input of key laboratory indicators by clinicians, and (2) image upload of laboratory reports, from which the system identifies and extracts item values and automatically matches them to a predefined list of medical indicators, greatly improving the efficiency and accuracy of data entry.

After the data input is completed, the system uses the trained XGBoost classification model to distinguish between eczema and psoriasis, and simultaneously outputs the corresponding prediction probability. The diagnostic results are presented through a combination of text and visual outputs, enabling clinicians to quickly interpret the model’s decision tendencies. This system integrates AI-based modeling, automated data collection, and a clinical interface, demonstrating the practical potential of intelligent auxiliary diagnosis for dermatological diseases in clinical settings. To further validate the usability of the web-based tool after deployment, we evaluated it using an external dataset comprising 469 patients from Shengjing Hospital, all of whom were pathologically diagnosed and excluded from the original model training. Among them, 343 cases were correctly classified by the model, yielding an accuracy of 73.13%.

## Discussion

In this multicenter retrospective cohort study, we select 14 features from clinical and serological indicators and developed eight machine learning models for the differential diagnosis of eczema and psoriasis. Among all candidate models, the XGBoost model achieved the best performance, with an AUC of 0.891 in the training set, 0.830 in the internl test set, and 0.812 in the external test set. These results indicate a strong classification ability in distinguishing between two common but frequently misdiagnosed inflammatory skin diseases in dermatology outpatient settings. To enhance the interpretability of the machine learning model, we further introduced SHAP analysis to assess feature importance in the XGBoost model. This approach helped reveal the specific contributions of key variables in the prediction process, thereby improving the clinical transparency and trustworthiness of the model. According to the SHAP analysis, the ten most influential variables among the 14 selected features were: dNLR, NeutCount, SIRI, RDW, EosCount, IgE, PDW, MonoCount, MCV, and WBC.

Overall, inflammatory markers were generally higher in the psoriasis group than in the eczema group (46, 47). Among them, dNLR, neutrophil count, and SIRI were identified as the top three most important features. Both dNLR and SIRI are neutrophil-based indices that reflect systemic immune activation. Previous studies have shown that these markers, particularly dNLR and SIRI, are significantly elevated in patients with psoriasis and may be associated with disease activity or severity (48). Neutrophils play a role not only in local inflammation but also in promoting systemic immune responses by releasing pro-inflammatory cytokines such as IL-17 and TNF-*α*, thereby contributing to the chronic and relapsing nature of psoriasis. In psoriatic lesions, neutrophil accumulation within the stratum corneum is commonly observed and may lead to the formation of Munro’s microabscesses. This classic histopathological feature, known for its high diagnostic specificity, reflects the ongoing infiltration of neutrophils into the epidermis (49). This local histological feature is consistent with elevated peripheral neutrophil counts and increased inflammatory ratios such as dNLR and SIRI, indicating systemic immune activation. Although tissue and blood neutrophil levels are not always linearly correlated, both represent distinct aspects of the inflammatory response and contribute to the overall inflammatory burden in psoriasis. In psoriasis patients, RDW was significantly elevated, which may be attributed to red blood cell dysregulation, chronic inflammation, or oxidative stress (50). PDW and MonoCount were also increased, indicating platelet activation and monocyte involvement in inflammatory signaling pathways (51). While WBC elevation is common across various inflammatory conditions and lacks disease specificity, it still ranked among the top ten important features in this study. Given that blood samples were collected during outpatient visits and most psoriasis patients were in the active stage of the disease, peripheral white blood cell counts likely reflect systemic inflammatory activity. WBC levels have been shown to correlate closely with disease activity in psoriasis, thereby contributing valuable discriminative power to the model. In contrast, eczema patients displayed more prominent characteristics in IgE, EosCount, and MCV. The rise in eosinophils indicates Th2-driven eosinophilic inflammation, highlighting an allergic background in eczema. Eosinophils can release various inflammatory factors, contributing to skin barrier damage and inflammation, playing a significant pathogenic role in chronic eczema. Their effects are not limited to local inflammation but may also influence systemic immune balance, promoting allergic reactions. Elevated IgE further supports the association between eczema and allergic constitution. As a key mediator of hypersensitivity, IgE levels are significantly increased in atopic dermatitis and other forms of eczema, closely correlating with disease severity, and thus has high importance in the model as a clinical biomarker. MCV was slightly higher in eczema, which may reflect abnormal red blood cell maturation or potential nutritional status differences under chronic inflammation. Chronic inflammation can affect bone marrow hematopoiesis through released cytokines, leading to increased red blood cell volume. Furthermore, eczema patients often have nutritional absorption issues or dietary restrictions, which could be another reason for the increased MCV. Deficiencies in key nutrients like vitamin B12 and folate can disrupt red blood cell maturation, leading to elevated MCV (52). Additionally, the variables ranked 11th to 14th were age, MPV, BasoPercent, and PCT. Age was slightly higher in the eczema group, which may reflect a broader age distribution or different patient characteristics since psoriasis primarily affects younger to middle-aged individuals. BasoPercent was relatively higher in the eczema group, though basophils constitute a small proportion in peripheral blood. As important cells mediating allergic inflammation, basophils release histamine, leukotrienes, and other inflammatory mediators, increasing vascular permeability and promoting inflammatory cell migration. Their activation in eczema patients may be related to Th2-driven immune responses, particularly in chronic or recurrent eczema, where basophil involvement exacerbates local skin inflammation and itching. The mild increase in BasoPercent suggests a potential regulatory role in eczema’s immune microenvironment, reflecting the involvement of Type I hypersensitivity and chronic allergic inflammation (53, 54). MCV, although primarily used to evaluate anemia types, may indirectly reflect systemic inflammation responses in inflammatory diseases, serving as an indicator of metabolic inflammatory processes. PCT has recently been recognized as closely related to chronic inflammation. Platelets in diseases like psoriasis can participate in the inflammatory response by releasing chemokines, regulating leukocyte adhesion, and activating endothelial functions. PCT may indirectly indicate platelet activation levels and their role in inflammatory cascade reactions, potentially contributing to the immune microenvironment of the disease (55). The prominent performance of these features not only reflects the potential differences in systemic inflammatory characteristics between eczema and psoriasis, but also provides valuable clues for further investigation into their distinct pathogenic mechanisms, including immune responses, inflammatory pathways, and disease progression. Moreover, the SHAP summary plots clearly visualized the directional impact and relative contribution of each feature to individual predictions, thereby improving the interpretability of the model and enhancing its credibility and applicability in clinical practice. Importantly, this study not only established multiple machine learning models with favorable performance but also translated the algorithmic output into a practical clinical tool. By deploying the model on a web-based platform and integrating OCR technology, we enabled users to enter data either manually or by uploading laboratory reports, significantly improving diagnostic efficiency. This approach is particularly suited for primary care settings and resource-limited environments, where it can facilitate rapid preliminary screening and assist frontline clinicians in differentiating between eczema and psoriasis to a certain extent.

Although the machine learning models developed in this study demonstrated favorable classification performance in both the training and independent external validation sets, and the data were sourced from multiple hospitals across different cities, indicating a certain degree of generalizability, several aspects still warrant further improvement. First, our study relied on retrospective data. Second, although measures such as cross-validation and early stopping were applied to minimize overfitting, the possibility of residual overfitting cannot be fully excluded. Therefore, the findings should be interpreted with caution. Future prospective studies utilizing larger datasets from broader geographic regions and diverse healthcare settings are warranted to validate the robustness of our results. Finally, the study relied on standardized hematological test results. However, in practical clinical settings, variability in testing protocols, instruments, and reference ranges across different laboratories may affect model performance. Future work could consider incorporating calibration mechanisms or center-specific adjustments to address inter-laboratory variability. In conclusion, this study demonstrates the feasibility and clinical potential of combining machine learning algorithms with SHAP interpretability techniques for the intelligent differential diagnosis of dermatological diseases. By closely aligning algorithm development with real-world clinical workflows, we have developed an accessible, objective, and efficient diagnostic support tool. This approach offers a new perspective for promoting precision and intelligence in dermatological diagnosis and holds promise for broader application in future disease classification and decision-support tasks.

## Conclusion

We developed a machine learning model for the differential diagnosis of eczema and psoriasis based on serum biomarkers and demographic features. Furthermore, an OCR-enabled web platform was constructed to deploy this model. By providing rapid, non-invasive diagnostic support, it can reduce diagnostic delays and improve care quality in primary care or resource-constrained environments. The platform can also be integrated with electronic health records (EHRs), helping streamline workflows and enhance clinical efficiency. Future research should validate the model in larger, prospective, multicenter cohorts to confirm its generalizability and robustness. In terms of practical applications, the web-based tool integrated with OCR technology could be deployed in outpatient settings to provide rapid, non-invasive diagnostic support. It also has potential to assist clinicians in primary care or resource-limited settings, where dermatology specialists may be scarce, and integration into hospital EHR systems could further streamline clinical workflows and reduce diagnostic delays.

## Data Availability

The raw data supporting the conclusions of this article will be made available by the authors, without undue reservation.
