# Supplementary material for: Differential diagnosis of eczema and psoriasis using routine clinical data and machine learning: development of a web-based tool in a multicenter outpatient cohort
Source: Front Med (Lausanne). 2025 Oct 17;12:1667794. doi: 10.3389/fmed.2025.1667794 (PMC12575206; doi:10.3389/fmed.2025.1667794)
Supplement: Supplementary file 1 [file Table_1.DOCX]

**Table S1.** Baseline characteristics of the training set and test set after feature selection.

|  | **Variables** | **Total**  **(n = 1014)** | **Training set**  **(n = 607)** | **Test set**  **(n = 407)** | **P value** |
| --- | --- | --- | --- | --- | --- |
| **Personal**  **history** | age | 38 (29, 54) | 37 (28, 53) | 41 (29, 57) | 0.07 |
| **Clinical**  **blood**  **tests** | WBC,  10^9/L | 6.74 (5.70, 8.15) | 6.80 (5.75, 8.11) | 6.69 (5.67, 8.18) | 0.41 |
|  | BasoPercent,% | 0.50 (0.30, 0.70) | 0.50 (0.30, 0.70) | 0.50 (0.30, 0.70) | 0.12 |
|  | NeutCount, 10^9/L | 4.10 (3.30, 5.10) | 4.10 (3.30, 5.00) | 4.00 (3.20, 5.20) | 0.37 |
|  | MonoCount, 10^9/L | 0.40 (0.30, 0.50) | 0.40 (0.30, 0.50) | 0.40 (0.30, 0.50) | 0.90 |
|  | EosCount, 10^9/L | 0.13 (0.08, 0.25) | 0.12 (0.08, 0.23) | 0.14 (0.08, 0.27) | 0.10 |
|  | MCV, fL | 90.00 (88.00, 93.00) | 90.00 (88.00, 93.00) | 90.00 (87.45, 93.00) | 0.52 |
|  | RDW, % | 13.00 (12.60, 13.50) | 13.10 (12.60, 13.60) | 13.00 (12.50, 13.40) | 0.30 |
|  | MPV, fL | 8.80 (8.10, 9.57) | 8.90 (8.00, 9.55) | 8.70 (8.10, 9.55) | 0.60 |
|  | PCT, % | 0.21 (0.18, 0.25) | 0.22 (0.18, 0.26) | 0.21 (0.19, 0.25) | 0.47 |
|  | PDW, % | 16.50 (16.10, 16.90) | 16.40 (16.00, 16.80) | 16.50 (16.10, 16.80) | 0.47 |
|  | IgE | 63.47 (23.70, 196.50) | 61.78 (23.75, 168.40) | 69.96 (23.73, 211.00) | 0.39 |
| **Derived**  **inflammatory**  **indices** | dNLR | 0.87 (0.83, 0.89) | 0.87 (0.83, 0.89) | 0.86 (0.83, 0.89) | 0.15 |
|  | SIRI | 0.88 (0.62, 1.34) | 0.87 (0.62, 1.33) | 0.91 (0.62, 1.35) | 0.92 |
